# Opening of Sanatorium and Convalescent Home, Heatherside, Camberley

**Published:** 1904-07-02

**Authors:** 


### HOSPITAL ADMINISTRATION.

### CONSTRUCTION AND ECONOMICS.

### OPENING OF SANATORIUM AND CONVALESCENT HOME, HEATHERSIDE, CAMBERLEY.

The sanatorium at Frimley, described in The Hospital for June 25th, was opened on that date by their Royal Highnesses the Prince and Princess of Wales, in the presence of over 500 visitors, who travelled by special train from Waterloo. The heavy storms of the early part of the afternoon cleared in time to allow the ceremony to take place in comfort out of doors, and punctually at 4 o'clock, the Royal motor arrived, the band of the 2nd battalion Royal Lancaster Regiment, which furnished the guard of honour, playing the National Anthem. The escort was formed by the Surrey (Princess of Wales) Imperial Yeomanry.

A raised daïs decorated with plants and flowers had been erected before the main entrance to the administrative block, and here their Royal Highnesses were received by Lieut.-General Sir John French, Commander First Army Corps, Mr. E. D. Stern, High Sheriff of Surrey, Major-General the Right Hon. Lord Cheylesmore, Chairman of the Committee of Management of the Brompton Hospital, who was accompanied by Lady Cheylesmore, and his Grace the Duke of Wellington, K.G., member of the Committee. General Sir Henry Trotter, Sir Charles Cust, R.N., and the Countess of Airlie were in attendance on their Royal Highnesses. The following were presented to the Prince and Princess by Lord Cheylesmore:—Mr. George Ste. Croix Rose (Vice-Chairman), Dr. C. Theodore Williams, Dr. J. Kingston Fowler, Dr. P. Horton-Smith, members of building committee; Mr. E. T. Hall, architect; Mr. W. H. Theobald, secretary; and Mrs. Price, lady superintendent, who presented the Princess of Wales with a bouquet of pink carnations, lilies of the valley, and orchids.

Lord Cheylesmore, in the unavoidable absence of the Earl of Derby, K.G., President of the hospital, then delivered an address of loyal welcome. The hospital, he said, had from its foundation in 1841 enjoyed the continuous patronage and support of the Royal Family, the foundation stone of the original hospital having been laid by the late Prince Consort, in 1844; that of the south or extension wing, in 1879, by his Majesty King Edward VII. as Prince of Wales, in the presence of the Queen (then Princess of Wales), Princess Helena, the late Duke of Cambridge, and Prince Christian of Schleswig-Holstein. The building which was to be opened was a further development of the work so long carried on at Brompton, and was intended for the open-air treatment of tuberculosis and for convalescent cases from the parent institution, accommodation being provided for 100 patients of both sexes, with the corresponding medical and nursing staff. It would entail an increased annual expenditure of some £10,000 for maintenance, but as it would be one of the first institutions of the kind for treating gratuitously on the open-air system the poorest classes from all parts of the country, the committee felt that it was entitled to the support of the public at large.

The committee, he said, wished to emphasise the fact that the treatment of consumption required the most liberal diet and cheerful surroundings, and although the annual cost per head in the Brompton Hospital might appear high, the committee, acting upon the advice of their medical staff, which comprised the most eminent authorities on the subject, were determined that nothing but want of financial support should induce them to depart from the principles in this respect which had hitherto prevailed in their institution.

In offering to their Royal Highnesses their most grateful thanks for honouring the occasion by their presence, and relying upon the blessing of the Almighty upon this new enterprise, the committee asked their Royal Highnesses to declare the building open as a sanatorium and convalescent home in connection with the Brompton Hospital.

Prayer was then said by the Bishop of Southampton, who was attended by the Rev. Wm. Bassett, Rector of Frimley, and the Rev. Henry Hall, Chaplain to the Brompton Hospital. The hymn, "All people that on earth do dwell" was sung, led by the choir of St. Peter's Church, Frimley, and H.R.H. the Prince of Wales made the following reply to the address:—

Lord Cheylesmore, Ladies, and Gentlemen,—The Princess and I thank you sincerely for your kind welcome. It gives us great pleasure to be present here to-day to assist in the inauguration of the Sanatorium and Convalescent Home of the Brompton Hospital.

We trust that our presence may be considered as evidence of a desire to preserve that connection which has existed between our family and the hospital ever since the first stone of the original building was laid by my grandfather, the Prince Consort, 60 years ago. We are also glad to be present at the opening of another institution for the open-air treatment of tuberculosis. For, although the death-rate from tuberculous affections in England and Wales has decreased, the dread disease is still one of the principal causes of death in the United Kingdom. Nor do the number of deaths from consumption, amounting to upwards of 60,000 a year, represent the terrible havoc which it produces in the population. For, alas! being a chronic disease, many persons pass a lingering existence through its different stages, and being mostly incapacitated from work, are a burden to their families and the State. And it was with an earnest desire to alleviate the sufferings of his thus afflicted subjects, that the King recently devoted a large sum of money, which was handed over to his Majesty for charitable purposes, to the founding of a home and sanatorium for the open-air cure. The aim of the treatment in this and similar institutions is to raise the standard of the resisting power of the patients by supplying them with abundant sunshine and fresh air on a dry soil, with good and suitable food, with exercise adapted to individual requirements, and with skilled medical supervision in daily life. This work, which has been so courageously undertaken by the authorities of the Brompton Hospital, will entail not only a large capital, but an increased annual expenditure for maintenance. Bearing in mind that its object is for the treatment of the poorest classes, I earnestly trust that it will meet with the hearty and liberal support of the public.

I have now much pleasure in declaring the buildings open, and the Princess and I join with you in the prayer that Divine Providence may bless this institution, and prosper the work of those now or ever connected with it.

The Prince of Wales was then presented by the architect with a special key to open the building, and accompanied by the Princess, and attended by the building committee, architect, secretary, and lady superintendent, entered and inspected some of the wards and rooms of the administrative block. Their Royal Highnesses subsequently signed the official record of the opening of the building, and placed their names in the visitors' book.

Before leaving, the Prince of Wales inspected the guard of honour, and was heartily cheered by the children of the Royal Albert Orphan Asylum, whose band played the National Anthem.

